# Impacts of longitudinal water curtain cooling system on transcriptome-related immunity in ducks

**DOI:** 10.1186/s12864-024-10179-4

**Published:** 2024-04-03

**Authors:** Qian Hu, Tao Zhang, Hua He, Fajun Pu, Rongping Zhang, Liang Li, Jiwei Hu, Lili Bai, Chunchun Han, Jiwen Wang, Hehe Liu

**Affiliations:** 1 Farm Animal Genetic Resources Exploration and Innovation Key Laboratory of Sichuan Province, Sichuan Agricultural University, 611130 Chengdu, Sichuan P.R. China; 2 Key Laboratory of Livestock and Poultry Multi-omics, Ministry of Agriculture and Rural Affairs, 611130 Chengdu, Wenjiang District, Sichuan P.R. China; 3 National Key Laboratory for Swine and Poultry Breeding, Wuhan, P.R. China

**Keywords:** Duck, Housing environment, Immune organs, Longitudinal water curtain cooling system, Transcriptome

## Abstract

**Background:**

The closed poultry houses integrated with a longitudinal water curtain cooling system (LWCCS) are widely used in modern poultry production. This study showed the variations in environmental conditions in closed houses integrated with a longitudinal water curtain cooling system. We evaluated the influence of different environmental conditions on duck growth performance and the transcriptome changes of immune organs, including the bursa of Fabricius and the spleen.

**Result:**

This study investigated the slaughter indicators and immune organ transcriptomes of 52-day-old Cherry Valley ducks by analyzing the LWCC at different locations (water curtain end, middle position, and fan cooling end). The results showed that the cooling effect of the LWCCS was more evident from 10:00 a.m. -14:00. And from the water curtain end to the fan cooling end, the hourly average temperature differently decreased by 0.310℃, 0.450℃, 0.480℃, 0.520℃, and 0.410℃, respectively (*P* < 0.05). The daily and hourly average relative humidity decreased from the water curtain end to the fan cooling end, dropping by 7.500% and 8.200%, respectively (*P* < 0.01). We also observed differences in production performance, such as dressing weight, half-eviscerated weight, skin fat rate, and percentage of abdominal fat (*P* < 0.01), which may have been caused by environmental conditions. RNA-sequencing (RNA-seq) revealed 211 and 279 differentially expressed genes (DEGs) in the ducks’ bursa of Fabricius and spleen compared between the water curtain end and fan cooling end, respectively. The Gene Ontology (GO) and Kyoto Encyclopedia of Genes and Genomes (KEGG) analysis of the two organs showed the DEGs were mainly enriched in cytokine-cytokine receptor interaction, integral component of membrane, Retinoic acid-inducible gene I (RIG-I)-like receptors (RLRs) signaling pathway, etc. Our results implied that full-closed poultry houses integrated with LWCCS could potentially alter micro-environments (water curtain vs. fan cooling), resulting in ducks experiencing various stressful situations that eventually affect their immunity and production performance.

**Conclusion:**

In this study, our results indicated that uneven distributions of longitudinal environmental factors caused by LWCCS would affect the dressed weight, breast muscle weight, skin fat rate, and other product performance. Moreover, the expression of immune-related genes in the spleen and bursa of ducks could be affected by the LWCCS. This provides a new reference to optimize the use of LWCCS in conjunction with close duck houses in practical production.

**Supplementary Information:**

The online version contains supplementary material available at 10.1186/s12864-024-10179-4.

## Background

With the rapid development of the modern poultry industry and highly intensive production management, a fully stacked cage-raising mode is widely used. This model offers several advantages, including enhanced and intensive processing of metabolic waste; a higher feed conversion rate (FCR) and reduced production costs; and effective monitoring of the health and production performance of individual poultry. However, there are challenges associated with a full-closed house, as the birds are exposed to several stressors, such as temperature, relative humidity, stocking density, immunological challenges, airborne contaminants, etc. [1].. High environmental temperature and moisture will induce heat stress in the birds by altering their thermoregulatory mechanisms [2]. Among the various airborne contaminants, ammonia (NH_3_) is considered the most harmful gas in modern poultry houses, and excessive exposure to NH_3_ can adversely decrease the growth performance of animals [3]. Additionally, stressors in poultry houses also affect the immunological function of birds, leading to a decrease in specific antibody titers and an increase in disease susceptibility [4]. Therefore, a longitudinal water curtain cooling system (LWCCS) is adopted to control temperature and relative humidity through the process of evaporative cooling [5].

The LWCCS can effectively control the temperature and humidity in the full-closed house. Studies have shown that during June to September, the LWCCS exhibited a more pronounced cooling effect compared to an ordinary fan, and the average temperature dropped by 3.820℃, 6.330℃, 4.200℃, and 2.270℃, respectively [6]. Some researchers have identified that the best wind speed after the water curtain is 1-1.500 ms^− 1^, which could ensure a cooling efficiency of about 60–70% [7]. However, the conventional method of longitudinal ventilation can cause uneven temperature distribution and the creation of a harmful gas environment across different heights and sections in the poultry house. In a study conducted by Zhao et al. (2011), it was discovered that the temperature gradient in the airflow direction within the greenhouse integrated with LWCCS was about 0.1086 °C/m [8]. Another study revealed that by implementing a stable cooling system by the opening Fan-Pad system, the hourly mean temperature and relative humidity within the greenhouse from pad to fan ranged between 20 and 27 °C, and 50–68%, respectively. And the air temperature from the Pad to the Fan increased approximately by 7 ºC [9]. All the above factors may affect the production performance and health of animals. Therefore, it is crucial to enhance the effectiveness of the LWCCS in modern farms, as it plays a significant role in shaping the development and future of the poultry farm. At the same time, uneven environmental changes resulting from LWCCS may alter animal immunities. Generally, It was believed that environmental stressors induced the activation of the hypothalamus-pituitary-adrenal (HPA) axis, leading to negative effects on the physicochemical properties of animal blood and immune function [10]. In birds, the bursa of Fabricius and spleen play vital roles in maintaining health and are sensitive to continuous changes in the housing environment, as the unique immune organ and the body’s largest immune organ, respectively. Shah et al. (2020) reported that excessive exposure to NH_3_ resulted in reduced size of the bursa of Fabricius and thymus index, increased nitric oxide (NO) content and inducible nitric oxide synthase (iNOS) activity, and led to inflammation [11]. Furthermore, It has been reported that ducks exposed to 75 ppm ammonia for 10 days had significantly lighter spleen weights compared to those exposed to 10 ppm ammonia for 10 days. Additionally, ammonia was found to have an impact on the composition of cecal microflora [12].

Previous studies on the LWCCS primarily focused on aspects such as the structure and parameters of the water curtain [9], its cooling effect [8], and system installation height [13]. In the present study, we have expanded upon this research by evaluating the comprehensive impact of LWCCS on environmental parameters, immune status, and production performance of meat ducks within a fully closed commercial meat duck house. This investigation aims to contribute new insights for optimizing the environmental control strategies associated with LWCCS deployment in fully enclosed housing systems.

## Results

### Environmental parameters from water curtain end to fan cooling end

**Fig. 1:**
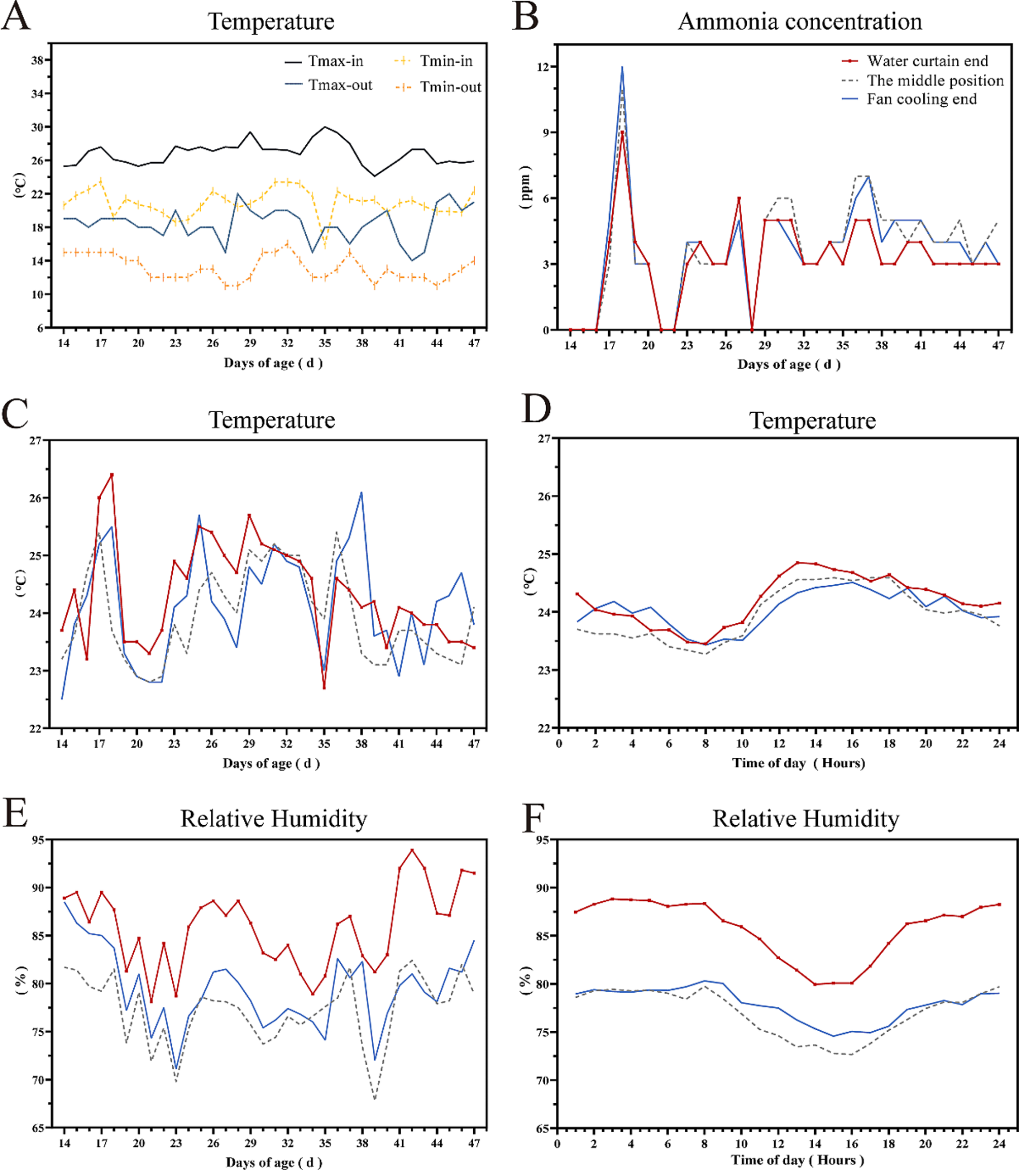
The graph shows the trend of environmental parameters. The horizontal axis represents the time, and the vertical axis represents the environmental parameters. (A) The trend of temperature changes inside and outside the duck house. Tmax-in and Tmin-in mean maximum and minimum temperature in the duck house, respectively. Tmax-out and Tmin-out mean maximum and minimum temperatures outside the duck house. (B) The trend of ammonia concentration in three positions in the duck house. Specifically, the ammonia concentration of 0 at 14, 15, and 16 days of duck age was due to the ventilation of the duck house. (C) The trend of temperature changes in three positions in the duck house from 14 to 47 days of age. (D) The trend of temperature change in the duck house for 24 h on different days. (E) The trend of relative humidity changes in three positions in the duck house from 14 to 47 days of age. (F) The trend of relative humidity changes in the duck house for 24 h on different days

**Fig. 2:**
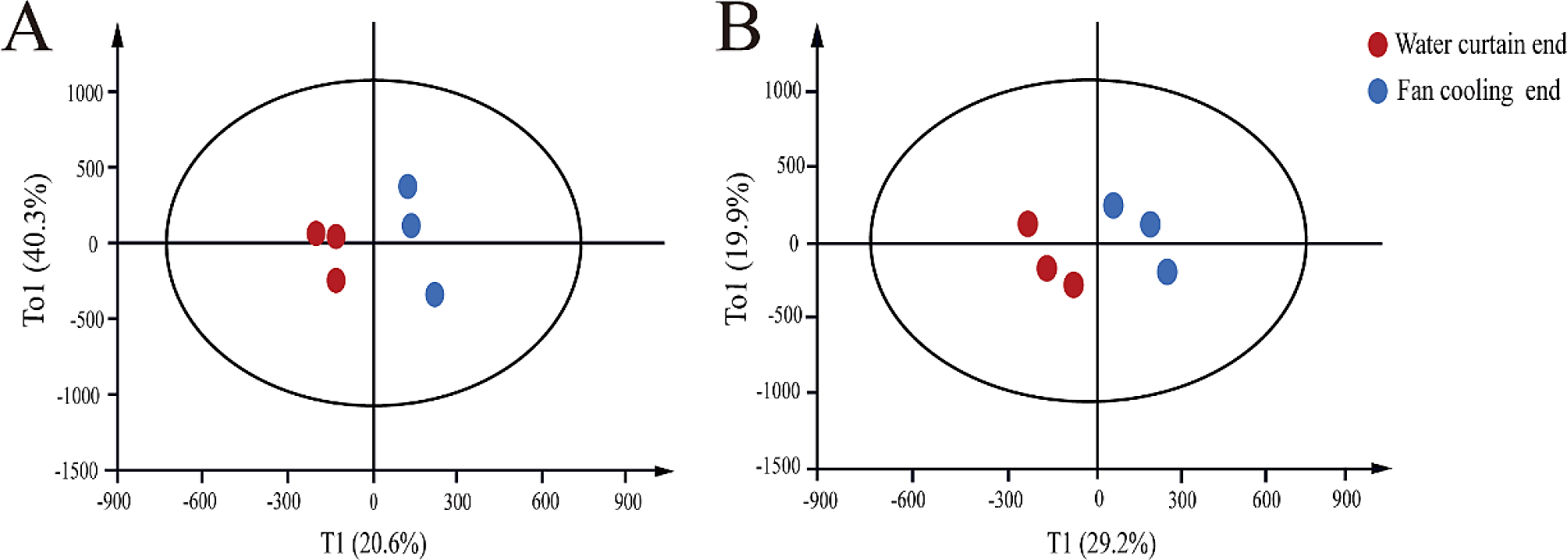
The PLS-DA model of the bursa of Fabricius (A) and spleen (B). Each point represents one sample, and the same color represents the same group. PLS-DA showed that PC1 of spleen transcripts accounted for 20.6% of the variation, and PC1 of bursal transcripts accounted for 29.2% of the variation, indicating that the samples were well clustered within the group, with good reproducibility and reliable sample quality

Firstly, we compared the temperature differences between the interior and exterior of the duck house. The results showed that, at 14–47 days old of ducks, the daily maximum and minimum indoor temperatures were differently higher than outdoors, with an average temperature increase of 8.360℃ and 7.840℃, respectively (*P* < 0.01) (Fig. 1A and Table S1). In the duck house, there was no difference in the daily average temperature and daily average ammonia concentration among the water curtain end, the middle position, and the fan cooling end from 14 to 47 days of duck age (Fig. 1B and C and TableS1). However, the ammonia concentration showed an increasing trend from the water curtain to the fan end. In addition, the cooling effect of LWCCS was evident from 10:00 a.m. -14:00. From the water curtain end to the fan end, the hourly average temperature differently decreased significantly, respectively (*P* < 0.05), with the falling temperature ranging from 0.310 to 0.520 °C, (Fig. 1D and Table S1). Similarly, the average relative humidity showed a distinct decrease from the water curtain end to the fan end, which dropped by 7.5% and 8.2% on a daily and hourly basis, respectively (*P* < 0.01) (Figs. 1E and 2F and Table S1).

### Body weight and carcass traits

We measured the individual body weight at 37, 43, and 52 days of duck age. At 37 days of age, there was no significant difference in the body weight of ducks among the three positions. At 43 and 52 days of age, the body weight of ducks differently increased from the water curtain end to the fan cooling end, and the average body weight increased by 0.1 and 0.7 kg, respectively (*P* < 0.05). However, the feed conversation ratio at 52 days of age gradually decreased from the water curtain to the fan cooling end (Table 1).

**Table 1:** Comparison of duck body weight among the three positions

Days of age	Water curtain end		The middle position		Fan cooling end	
	N	BW	N	BW	N	BW
37	161	2.280 ± 0.250^a^	159	2.260 ± 0.260^a^	159	2.220 ± 0.240^a^
43	160	2.530 ± 0.290^a^	158	2.640 ± 0.300^b^	158	2.630 ± 0.290^b^
52	115	2.500 ± 0.300^a^	145	2.590 ± 0.320^a^	154	3.200 ± 0.340^b^
CV	115	12.000%	145	12.360%	154	10.620%
FCR	115	2.700	145	2.640	154	2.100

Note: Values are mean ± standard error; CV means the coefficient of variation at 52 days of age; ^a−b^ means in each column not sharing a common superscript are significantly different (*P* < 0.05); FCR means feed conversation ratio at 52 days of age. N represents the number of ducks at different days of age. BW represents the body weight of duck

In terms of production performance, there were more differences observed among the three positions in all the 21 determined parameters, including dressed weight, half-eviscerated weight, skin fat rate, etc. (*P* < 0.01). Compared to the water curtain end, the fan cooling end had a significant increase in the dressed weight(*P* < 0.01), half-eviscerated weight, breast muscle weight, skin fat rate, and abdominal fat rate. In particular, the bursa of the Fabricius index and spleen index showed a distinct increase by 0.020% and 0.010%, respectively, in the fan cooling end compared to the water curtain end (*P* < 0.05) (Table 2).

**Table 2:** Comparison of the slaughter performance of ducks among the three positions

Items	Water curtain end	The middle position	Fan cooling end
Dressing weight (g)	2054.550 ± 236.150^Aa^	2088.500 ± 256.040^Aa^	2486.610 ± 210.930^Bb^
Dressed percentage (%)	80.740 ± 3.240^Aa^	81.140 ± 3.050^Aa^	77.110 ± 2.860^Bb^
Half eviscerated weight (g)	1945.050 ± 222.930^Aa^	1986.440 ± 229.860^Aa^	2341.270 ± 200.290^Bb^
Half eviscerated yield (%)	76.440 ± 3.210^Aa^	77.230 ± 2.670^Aa^	72.600 ± 2.670^Bb^
Eviscerated weight (g)	1805.660 ± 206.510^Aa^	1847.880 ± 213.740^Aa^	2169.610 ± 189.810^Bb^
Eviscerated percentage (%)	70.980 ± 3.250^Aa^	71.850 ± 2.780^Aa^	67.280 ± 2.800^Bb^
Breast muscle weight (g)	122.870 ± 31.390^Aa^	133.320 ± 32.260^Aa^	164.790 ± 25.270^Bb^
Breast muscle percentage (%)	13.450 ± 2.310^a^	14.290 ± 2.400^ab^	15.160 ± 1.560^b^
Breast brisket (mm)	9.820 ± 2.230^a^	10.060 ± 2.410^a^	11.960 ± 2.620^b^
Leg muscle weight (g)	116.210 ± 15.820^Aa^	126.540 ± 15.630^Aa^	140.100 ± 21.850^Bb^
Leg muscle percentage (%)	12.890 ± 1.240^a^	13.730 ± 1.160^a^	12.930 ± 1.660^a^
Skin fat (g)	330.050 ± 67.610^Aa^	327.380 ± 70.390^Aa^	446.770 ± 69.270^Bb^
Percentage of skin fat (%)	18.140 ± 1.910^Aa^	17.570 ± 2.120^Aa^	20.550 ± 2.400^Bb^
Abdominal fat (g)	19.630 ± 11.890^a^	18.320 ± 9.760^a^	26.930 ± 6.610^b^
Percentage of abdominal fat (%)	1.050 ± 0.580^a^	0.950 ± 0.440^a^	1.230 ± 0.290^a^
Heart (g)	12.600 ± 1.820^Aa^	12.730 ± 2.040^Aa^	15.600 ± 1.800^Bb^
Liver (g)	50.290 ± 7.100^Aa^	50.140 ± 8.540^Aa^	66.610 ± 21.130^Bb^
Muscular stomach (g)	49.630 ± 8.950^a^	49.160 ± 8.180^a^	56.430 ± 9.620^b^
Glandular stomach (g)	6.510 ± 1.550^Aa^	6.220 ± 1.050^Aa^	8.340 ± 2.940^Bb^
Spleen (g)	1.190 ± 0.400^Aa^	1.110 ± 0.370^Aa^	1.900 ± 0.970^Bb^
Spleen index (%)	0.050 ± 0.010^a^	0.040 ± 0.010^a^	0.060 ± 0.030^b^
Bursa of Fabricius (g)	0.940 ± 0.420^Aa^	1.170 ± 0.420^Aa^	1.850 ± 0.330^Bb^
Bursa of Fabricius index (%)	0.040 ± 0.010^Aa^	0.050 ± 0.020^Aa^	0.060 ± 0.010^Bb^

Note: Values are mean ± standard error. The column not sharing a common lowercase letters superscript represents significantly different (*P* < 0.05), while not sharing a common capital letters superscript is significantly different (*P* < 0.01)

### Quality control of RNA-seq data

Paired-end sequencing was performed with HiSeTM 2000 (Illumina, USA), and after quality control, an average of 24,362,926 and 23,109,980 clean reads were obtained from the bursa and spleen tissues, respectively. The Q30 value was higher than 90% for each sample, indicating that the sequencing quality was good and could be used for subsequent analysis (Table S2).

Partial least squares discriminant analysis (PLS-DA) was conducted using Simca software to analyze the bursa of Fabricius and the spleen of the six ducks. In order to be considered a significant biological model, the expected R^2^ and Q^2^, which are highly dependent on their application model, should be more than 0.500 and 0.400, respectively (Boulesteix [14]). In our established model, the values of R^2^ and Q^2^ meet these requirements (Table S2). This suggested that the samples were clustered well within the group, indicating good repeatability and reliable quality (Fig. 2A&B).

### Screening differentially expressed genes (DEGs)

**Fig. 3:**
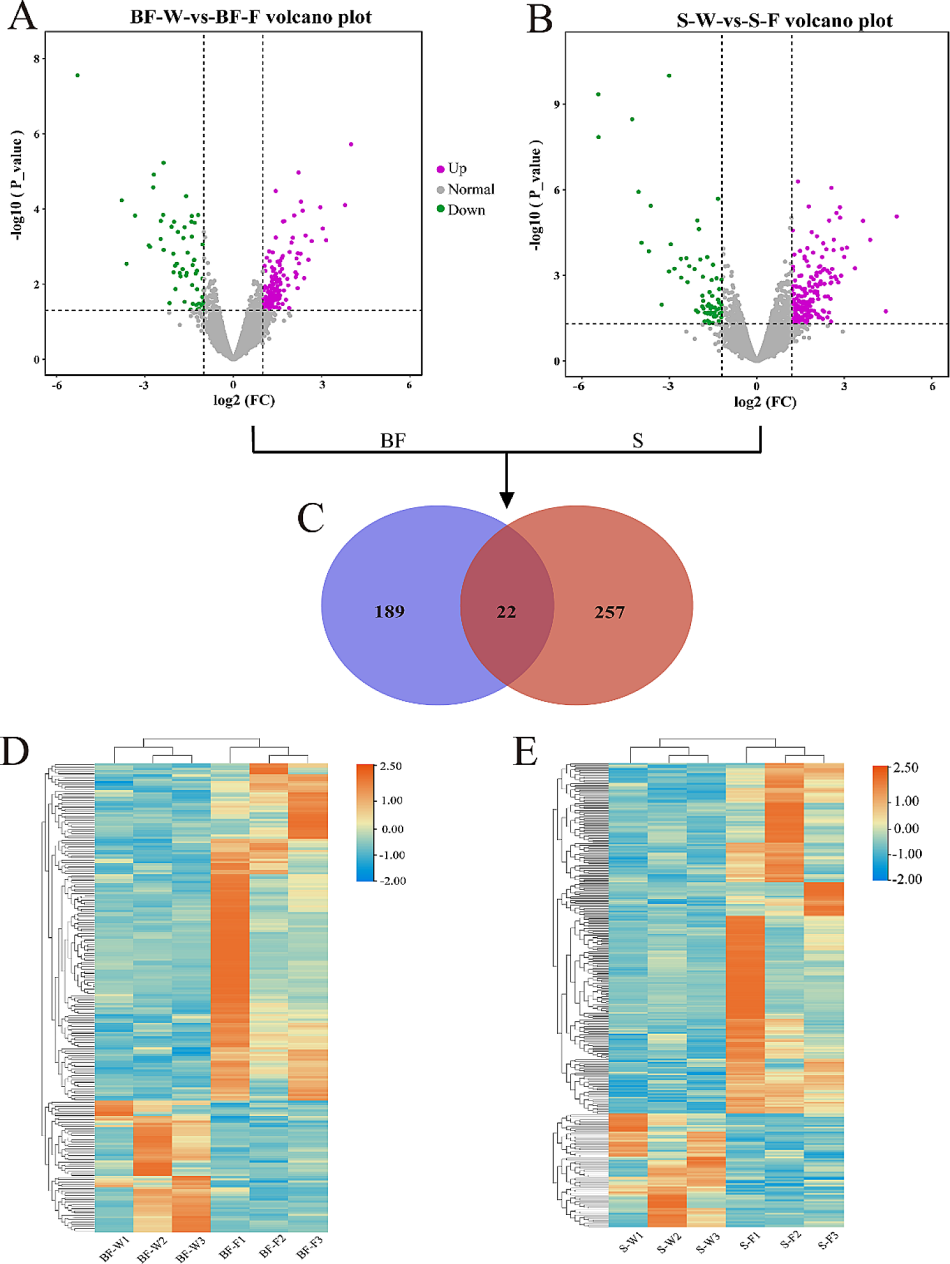
Volcano maps, Venn diagrams, and cluster heat maps of differentially expressed genes. BF and S represent the bursa of Fabricius and the spleen, respectively. W and F represent the wet curtain end and fan cooling end, respectively. (A) Volcano map of DEGs for the bursa of Fabricius. (B) Volcano map of DEGs for the spleen. Each point in the differential expression map represents a gene. The red dots represent down-regulated genes, the blue dots represent up-regulated genes, and the black dots represent non-differentially expressed genes. (C) Venn map of DEGs in the bursa of Fabricius and spleen. The green area represents the DEGs in the bursa of Fabricius. The red area represents the DEGs in the spleen. The cross-section represents the DEGs shared by the two tissues, and the number represents the number of DEGs. (D) Cluster analysis of DEGs in the bursa of Fabricius tissue from the two groups using CPM values. (E) Cluster analysis of DEGs in spleen tissue from the two groups using CPM values. The rows and columns represent genes and samples, respectively. Red denotes high-expression genes, and blue denotes low-expression genes. The closer the two-sample branches are, the more similar the gene’s expression pattern and the closer the trend

The DEGs were identified between the water curtain and fan cooling groups of the two tissues under the criteria of|Log_2_FC|≥1, with a *P*-value < 0.05. Within the DEGs, a total of 211 genes were identified in the bursa of Fabricius, which included 152 up-regulated and 59 down-regulated genes between the water curtain and the fan cooling groups (Fig. 3A). Additionally, a total of 279 genes were detected as DEGs in the spleen tissue, including 211 up-regulated and 68 down-regulated transcripts (Fig. 3B). Furthermore, 22 co-expressed differential genes were obtained in the two groups of the bursa of Fabricius and spleen tissues, including 7 annotated genes and 15 unannotated genes (Fig. 3C). Cluster analysis of the DEGs of the two tissues showed that genes with the same expression pattern were in the same group (Fig. 3D&E).

### Functional enrichment analysis

**Fig. 4:**
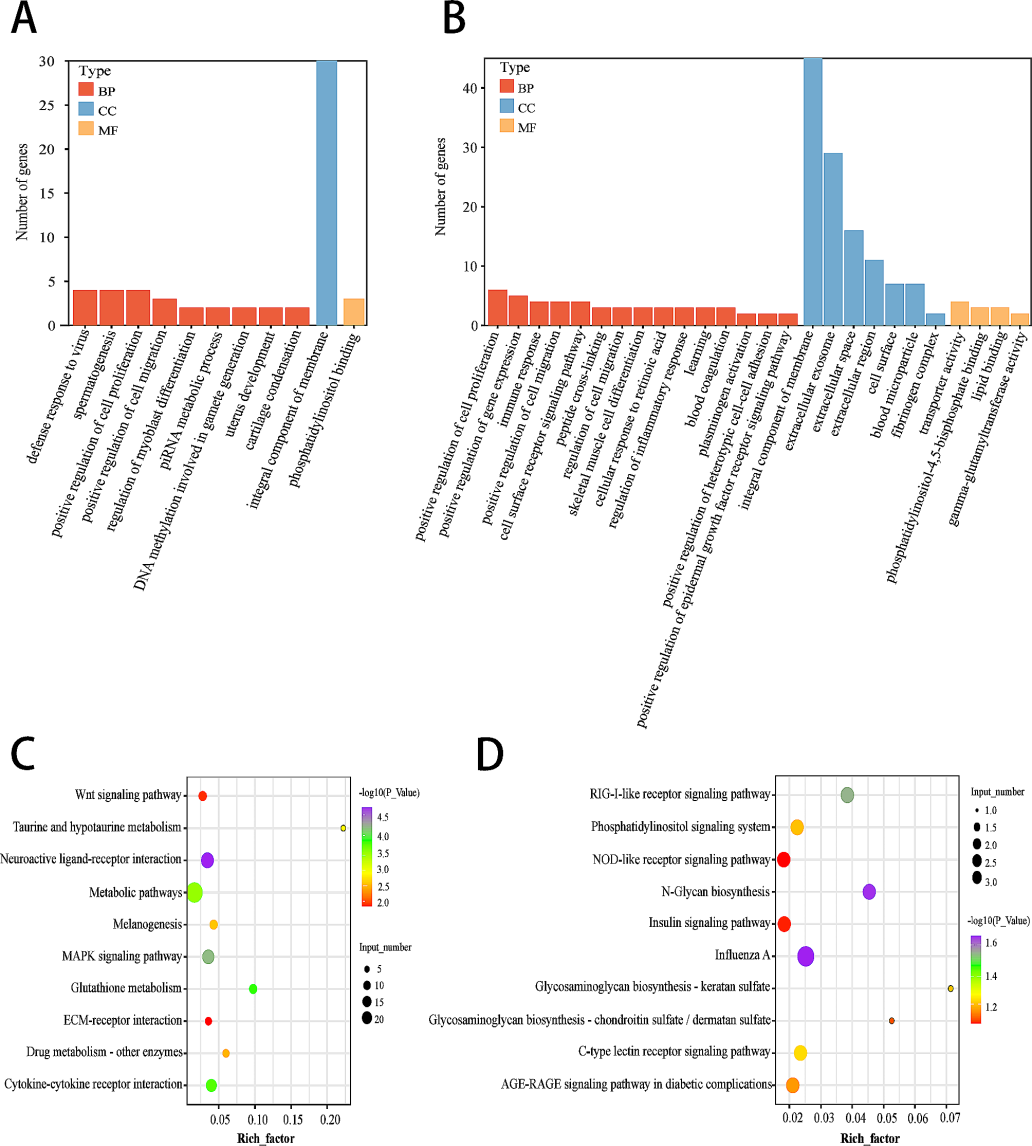
Q2

We performed a GO analysis of DEGs identified in both the bursa of Fabricius and the spleen to compare the functions of DEGs between the water curtain end and the fan cooling end. According to the David website, DEGs were divided into three main GO categories: biological process, molecular function, and cellular component. The results showed 11 GO terms in the bursa of Fabricius (Fig. 4A), and 26 GO terms in the spleen (Fig. 4B). Notably, the top significant GO terms in both immune organs were defense response to the virus (GO:0051607), cell surface receptor signaling pathway (GO:0007166), integral component of membrane (GO:0016021), the extracellular exosome (GO:0070062), and extracellular space (GO:0005615). In particular, the defense response to the virus plays an important role in binding immune active substances in cells and immune receptors on the cell surface.

Moreover, KEGG enrichment analysis was performed for all DEGs in the two tissues. The top 10 KEGG pathways in the bursa of Fabricius (Fig. 4C) showed that the most significant pathway was influenza A (apla05164), and the RIG-I-like receptor signaling pathway (apla04622), which was also related to immunity was also included. Similarly, the top 10 significantly enriched KEGG pathways of the spleen tissue (Fig. 4D) included neuroactive ligand-receptor interaction (apla04080), MAPK signaling pathway (apla04010), cytokine-cytokine receptor interaction (apla04060), all of which are related to immune response.

### Immune-related genes analysis

**Fig. 5:**
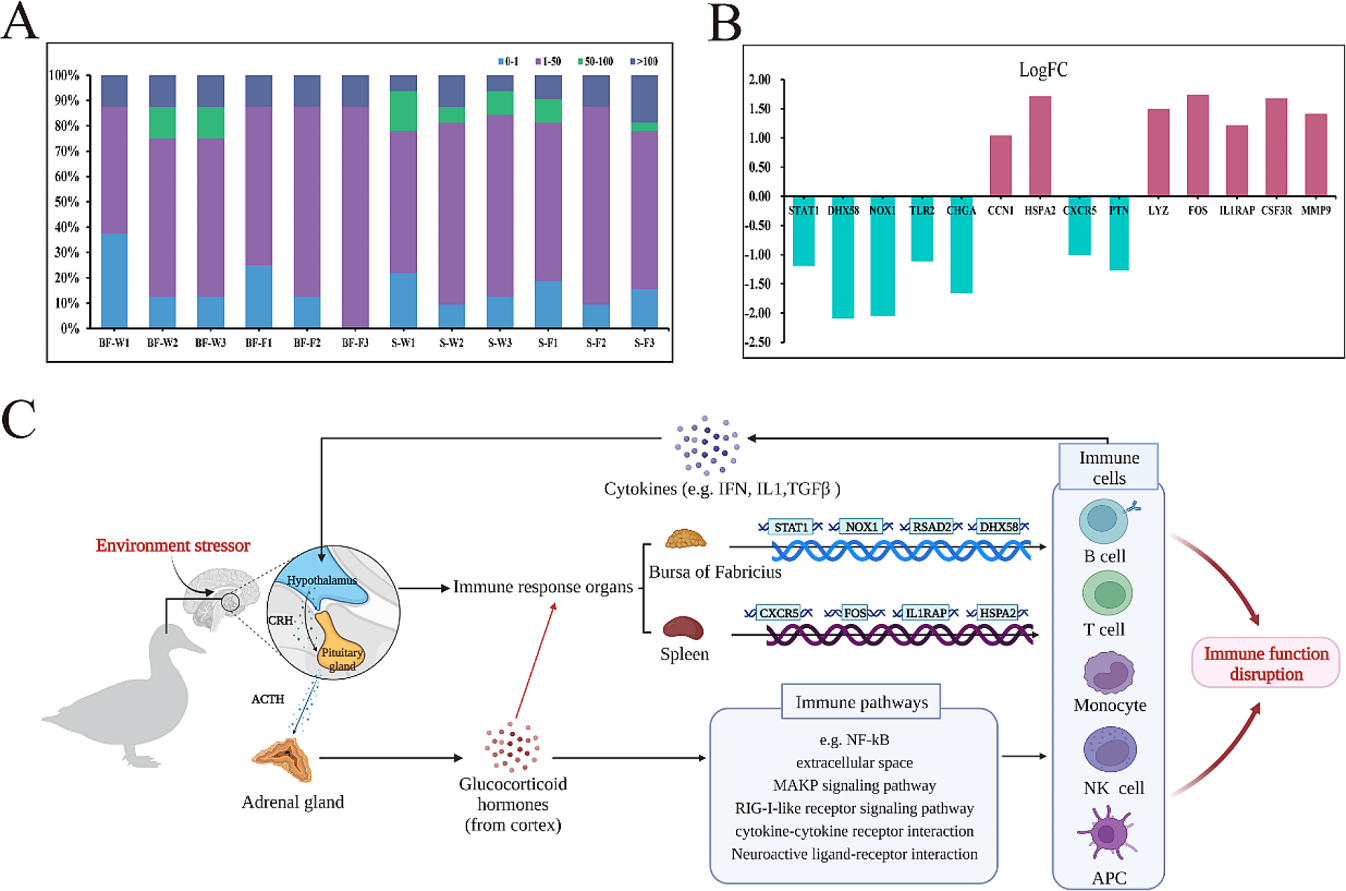
Environment stress-associated modulation of the HPA axis and immune response by the central nervous system. (A) Histogram of CPM value distribution of all immune-related DEGs. (B) Histogram of log (Fold change) value distribution of immune-related DEGs with high-level expression. (C) The process of the immune response. HPA, hypothalamic-pituitary-adrenal gland. ACTH, adrenocorticotropic hormone; CRH, corticotropin-releasing hormone; IFN, interferon; IL, interleukin; TGFβ, transforming growth factor beta. Additionally, the black arrows represent activation, and the red arrows represent inhibition

To illustrate the changes in the immune function of the bursa and spleen, all immune-related genes in ducks were sorted. A total of 40 immune-related DEGs were identified. There were 8 immune-related genes in the bursa of Fabricius, and 32 immune-related genes in the spleen, and most of the immune-related genes in the two organs were up-regulated at the fan cooling end (Tables 3and Table S3). Among them, 14 genes (*STAT1*, *DHX58*, *NOX1*, *TLR2*, *CHGA*, *CCN1*, *HSPA2*, *CXCR5*, *PTN*, *LYZ*, *FOS*, *IL1RAP*, *CSF3R*, and *MMP9*) were identified with high-level expression (CPM > 50 and *P* < 0.05; Table S3). Specifically, these immune genes were super-expressed in the two organs (CPM > 100), including *STAT1*, *HSPA2*, *CXCR5*, *LYZ*, and *CSF3R*. Figure 5A**&B** showed histograms and Fold changes based on the read count of these genes.

Furthermore, these genes were enriched in the RIG-I-like receptor signaling pathway, cytokine-cytokine receptor interaction, MAPK signaling pathways, etc. Some vital immune tissues of the immune system and many types of immune cells, such as the bursa, spleen, T and B lymphocytes, etc., play a role in humoral and cellular immunity. In our results, environment stress activated the HPA axis, regulated by these genes (*STAT1*, *NOX1*, *DHX58*, and *RSAD2*), and B lymphocytes matured and migrated in response to immune stress in the bursa of Fabricius (Fig. 5C**)**. The spleen was involved in the T-cell immune response, and gene regulatory proteins such as *HSPA2* and *IL1RAP* were connected to receptors to activate signaling pathways such as MAKP and NF-kB to induce the production of various immune cytokines (Fig. 5C). We speculated that environmental changes might affect immune activity through these genes, thus causing changes in duck production performance and health status.

**Table 3:** The statistics of immunity genes detected in the spleen and the Bursa of Fabricius of ducks

Items	Immune-related DEGs	Gene name
Bursa of Fabricius	8	*STAT1*|*DHX58*|*FGFRL1*|*RSAD2*|*NOX1*|*CHGB*|*BMPR1B*|*TNFRSF17*
Spleen	32	*POMC*|*CGA*|*CCN3*|*TLR2*|*OSTN*|*CHGA*|*CCR6*|*BPIFC*|*GH1*|*CD4*|*CCN1* |*HSPA2*|*CXCR5|PTN*|*LYZ*|*FOS*|*IL1RAP*|*CSF3R*|*NTS*|*FGF18*|*IFITM1*| *IL18RAP*|*VIPR2*|*RBP4*|*VEGFD*|*APOH*|*ADIPOQ*|*MMP9*|*NR1H4*|*TG*| *FABP3*| *SLIT2*

## Discussion

Nowadays, more attention has been paid to the effect of LWCCS on poultry and livestock houses’ environment with the rise in popularity of caged-housing mode. The main environmental factors inside the housing environment are air temperature (T), relative humidity (RH), ammonia concentration (NH_3_), etc. The LWCCS is adopted to help control the detrimental impact of these environmental factors on the well-being and productivity of poultry. Some studies reported that from the water curtain to the fan cooling end, the temperature and relative humidity decreased while the ammonia concentration increased inside the poultry house [13], which was consistent with our results. The water curtain end was the air inlet, while the fan end was the air outlet. Due to the longitudinal ventilation, the dirty air was blown to the back end, accumulating the contaminated air at the fan end. The effective design and management of livestock and poultry housing are directly correlated with production performance and contribute significantly to the maintenance of the health of the animal’s immune systems. A well-designed housing system supports optimal conditions that enhance feed efficiency, stimulate growth, and mitigate the spread of diseases [15]. Factors such as indoor air quality, ventilation systems, and overall hygiene directly impact the immune functionality of livestock and poultry. Suboptimal housing conditions may induce stress and fatigue, consequently diminishing the efficacy of the immune system and elevating the risk of diseases [16].

The production performance level reflects poultry’s environmental comfort and health status. In terms of production performance, the results showed that dressed weight, half eviscerated yield, and other slaughter performance parameters and pre-slaughter live weight at the water curtain end were significantly lower than those of the fan cooling end. We speculated that this might be due to the higher temperature and humidity at the water curtain end than the fan cooling. Studies have reported that the optimal ambient temperature for meat ducks is 16–22 °C, within which their best production performance could be achieved [17]. When the ambient temperature is higher than the appropriate temperature, poultry feed intake would be reduced. The earlier studies reported that when the environmental temperature is 27 ℃, the high humidity may reduce the feed intake of broilers and slow the growth of broilers; at 29 ℃, RH increases from 40 to 80%, and the feed intake and production performance of broilers decrease significantly [18–20]. Our result was consistent with these earlier findings. Furthermore, poultry production generated many harmful gases such as ammonia, methane, and nitrogen oxide, which affected living animals’ health in a closed house ( [19, 21, 22]. In our study, the meat duck’s bursa and spleen weight at the fan cooling end was significantly higher than at the water curtain end. The poor housing environmental conditions could negatively impact the immune response of poultry. These results indicated that meat ducks have adapted to resist the harsh environment with higher ammonia concentration at the fan cooling end.

Based on this, the impact of environmental factors on the immune organs can be used as a new perspective to evaluate LWCCS, in addition to evaluating changes in environmental parameters and duck production performance. As immune organs, the bursa of Fabricius and the spleen play a significant role in immunoreaction. The bursa of Fabricius provides a microenvironment for the differentiation and development of B lymphocytes, which dominate the body’s humoral immunity after maturation [23]. The immune function of the spleen is mainly divided into three parts: hematopoiesis, filtration, and cellular immune response [24]. In the current study, gene expression in the spleen and bursa of Fabricius was determined by RNA-seq. A total of 279 and 211 DEGs were identified in the spleen and the bursa of Fabricius, respectively. Upon analysis of gene ontologies, the DEGs were enriched in immune-related GO terms related to the integral component of the membrane and the extracellular exosome. Pathways analysis showed that the DEGs were mainly enriched in cytokine-cytokine receptor interaction and RIG-I-like receptor signaling pathways, which are all related to immune activities. Previous studies have shown that inappropriate temperature, humidity, and high ammonia concentration decreased the immune response of broiler chickens in closed houses [25, 26]. LWCCS could cause uneven distribution of the environment in the duck house. Therefore, this suggests that LWCCS impacts the immune function of the Fabricius and the spleen.

To further illustrate how LWCCS affected the function of the spleen and bursa of Fabricius, the expression of immune-related genes in ducks’ spleen and the bursa of Fabricius were investigated. The current study revealed 40 immune-related genes differentially expressed under the influence of different positions of LWCCS. Among these genes, *CD4* played an essential role in the immune response and served multiple functions in responses against both external and internal threats. Specifically, in T-cells, the *CD4* gene acts as a coreceptor for MHC and, in conjunction with the T-cell receptor (TCR), activates T-helper cells [27]. In contrast, the *FOS* gene activated TGF-β-mediated signaling at the AP1/SMAD-binding site, which may suppress T cell differentiation [28]. *IL1RAP* gene regulated inflammatory response through the NF-kB signaling pathway [12]. In addition, it was worth noting that most of the DEGs were up-regulated. This suggested that the poor environmental conditions at the fan cooling end promoted ducks’ immune response, although the specific mechanism needs further study.

## Conclusions

Our study highlights the significant impacts of the Longitudinal Water Curtain Cooling System (LWCCS) on duck production. LWCCS-induced uneven environmental factors notably affect key production metrics such as dressed weight, breast muscle weight, and skin fat rate. Additionally, LWCCS alters the expression of immune-related genes in duck spleens and bursa. These findings stress the importance of integrating environmental considerations into duck production optimization strategies. Understanding duck responses to LWCCS informs management practices to enhance production efficiency and animal welfare. In summary, our results offer insights for integrating LWCCS into closed duck house systems, advancing practical production methods in the duck farming industry.

## Materials and methods

### Ethics approval

All animal handling procedures were approved by the Institutional Animal Care and Use Committee (IACUC) of Sichuan Agricultural University (Chengdu campus, Sichuan, China, Permit No. DKY20170913).

### The model of the fully closed duck house, experimental design, and sampling

**Fig. 6:**
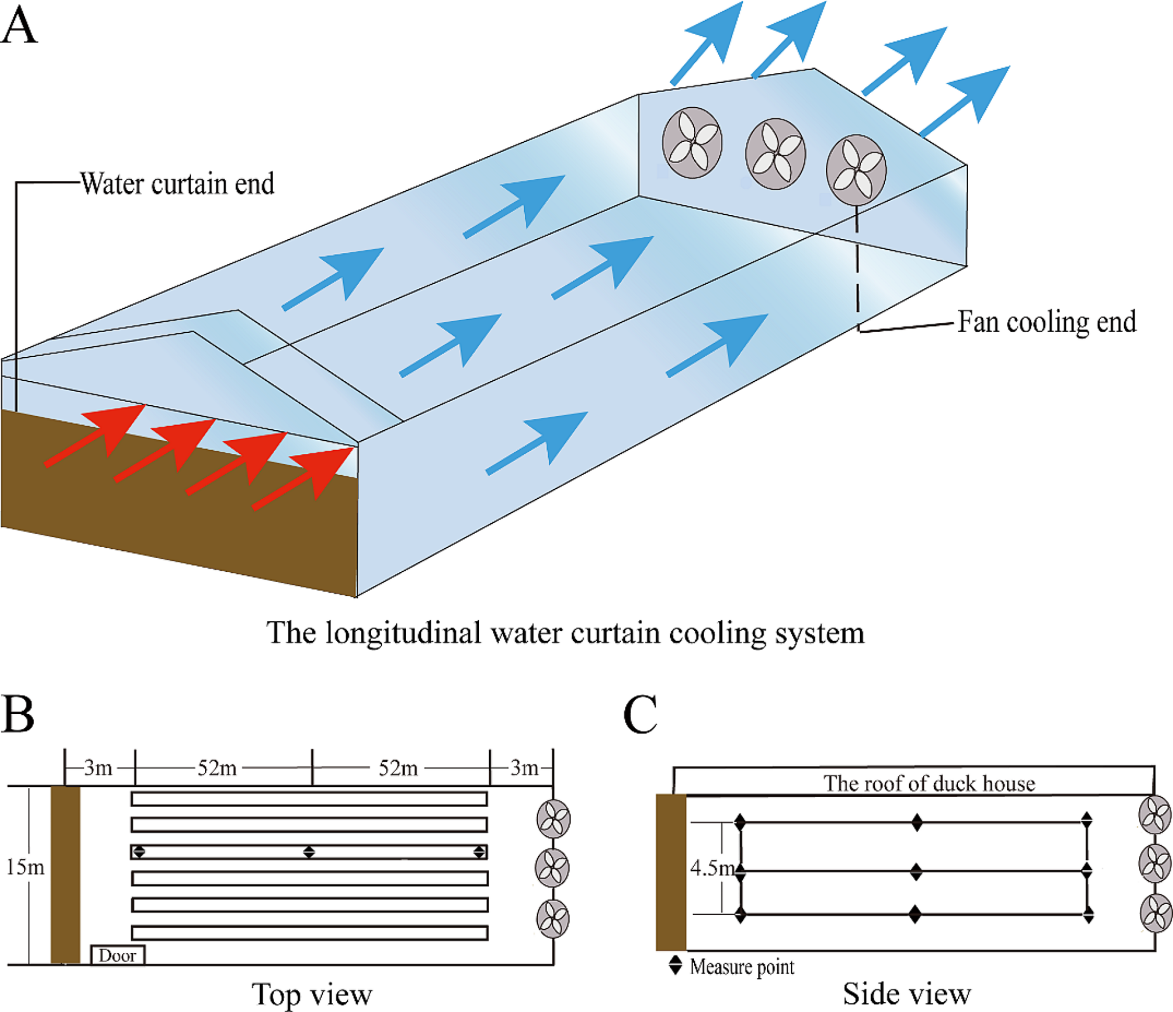
Schematic diagram of the experimental duck house

The duck house environment regulation system with the overall framework diagram is provided in Fig. 6A. The duck house used in this study was a fully enclosed standardized structure measuring a length of 110 m, a width of 15 m, and a height of 4.5 m. The roof of the duck house was made of color steel tile, the ceiling was constituted of plastic pinch plate, and the wall was made of brick-concrete structure. We adopted the water curtain filtration and negative-pressure ventilation method to control the temperature and humidity of the duck house. There were 18 negative-pressure exhaust fans at the air outlet end of the wall under the adoption of the converter drive. Additionally, we constructed a water curtain, made of a tile stack structure, spanning an area of 8.8 m^2^ at the air inlet end of the house wall.

The Cherry Valley commercial ducks were provided by Sichuan New Mianying Agriculture and Animal Husbandry Co. Ltd (Mianyang, China). A total of 486 ducks were divided into 3 groups (water curtain end, the middle position, fan cooling end), and each group consisted of 9 cages (3 floors and 3 adjacent cages on each floor). Each cage had 18 ducks (9 males and 9 females). The feed intake, light intensity, feeding pattern, and temperature were kept consistent during the experiment. All ducks were reared in cages (2.0 × 1.3 × 0.7 m) in a temperature- and humidity-controlled breeding house. From 14 to 43 days of age, the ducks had free access to drinking water and feed. However, from 44 to 51 d of age, their feeding was restricted to a daily limit of 120 g, based on the nutritional information of the diet provided in Supplementary Table 4. Each group was exposed to white light-emitting diodes (LED) for 24 h (24 L:0 D).

### Environment measurement

The fully closed duck house has six rows of duck hutches with three layers. The fourth row of cages near the entrance of the duck house was 3 and 55 m away from the water curtain end and 3 m away from the fan cooling end. We used the temperature and humidity sensors and the ammonia concentration sensors to monitor the environmental information constantly within the duck house (Fig. 6B and C). Air temperature and relative humidity inside the closed duck house were measured every 10 min using an automatic temperature and humidity data logger monitor (TH20R-EX, Miaoguan Technology Co. Ltd, Pingyang County, China). We calculated hourly average temperature and relative humidity values in the duck house for 24 h from 14 to 47 days of age, respectively. Moreover, a portable gas detector measured the ammonia concentration in the same area daily at 9–10 a.m. (ADK S-4, EDKORS Instruments Co. Ltd, Changzhou, China). The ammonia concentration was measured three times a day, and then the daily average value was taken as the final measurement.

### Measurements on growth performance and carcass traits of ducks

The weights of the ducks were measured at different time points; 1, 13, 37, 43, and 52 days. Randomly selected ducks that were 1-day-old and 13-day-old ducks were weighed, and the average weight of the whole group was recorded. After 13 days of age, each duck was individually weighed at three time points: 37, 43, and 52 days of age. According to the standard (NYT823-2004 poultry production performance terminology and statistical measurement methods), 54 ducks (comprising of 3 groups with 27 males and 27 females) were selected for the slaughter performance test at 52 days old. The selected experimental ducks were killed by inhaling carbon dioxide and cervical dislocation after fasting for about 12 h. The slaughter performance, including dressed carcass, eviscerated carcass, and the half-eviscerated carcass, thigh, breast, abdominal fat, skin fat, heart, liver, spleen, bursa, thymus, and proventriculus, were weighed accordingly. After ducks were weighed and slaughtered, RNA samples of the bursa of Fabricius and spleen were collected, initially stored in liquid nitrogen, and then transferred to an ultra-low temperature refrigerator at -80℃.

### Total RNA extraction and RNA-seq

The RNA samples were extracted from the bursa of Fabricius and the spleen tissues of 3 male ducks, each from two different groups: the water curtain end and fan cooling end at 52 days of age. Total RNA was extracted from tissues using Trizol reagent (Invitrogen, CA, USA) according to the manufacturer’s instructions. The degree of RNA degradation was analyzed by agarose gel electrophoresis, and RNA purity was detected using a Nanodrop 2000 spectrophotometer (Thermo Fisher Scientific, Wilmington, USA). Then, mRNA was purified from total RNA using poly-T oligo-attached magnetic beads, and a fragmentation buffer was utilized for RNA fragmentation and generated short RNA strands. Subsequently, the first cDNA strand was synthesized using random hexamer primers and RNA fragments as a template. The second-strand cDNA was synthesized using RNase H and DNA polymerase I. Short cDNA fragments were purified with AMPure XP Beads, then a terminal repair was done, poly (A) was added, and an adapter was implemented. PCR amplification was finally performed, and PCR products were purified with AMPure XP beads to yield the final library. The library quality was assessed on the Agilent Bioanalyzer 2100 system (Agilent, USA). The library preparations were sequenced on an Illumina NovaSeq 6000 platform, and the sequencing reading length was PE150.

### RNA-seq data analysis

High-throughput sequencing data was originally presented as raw image data files. After base recognition by bcl2fastq Conversion Software v1.8.4 (Illumina, USA), it was converted into raw data. In the quality control step, clean reads were obtained by removing those containing adapter, poly-N, and low-quality reads from the data. All downstream analyses were based on clean data with high quality. The clean data were matched to the reference genome of the Peking duck (ZJU1.0, https://www.ncbi.nlm.nih.gov/assembly/GCF_015476345.1) by using Hisat2 v2.1.0 [29]. The transcripts were assembled using Stringtie v1.3.3b [30], and the genes were quantified. The differentially expressed genes (DEGs) between the water curtain group and the fan cooling group were identified by the edgeR package. The screening criteria were *P*-value < 0.05 and|Log_2_ (fold change)| >1. Gene expression levels were quantified using CPM.

### Gene ontology and KEGG pathway analysis

Gene ontology (GO) enrichment analysis of differentially expressed genes was performed using the David website (https://david.ncifcrf.gov/). Pathway enrichment analysis was assessed using the Kobas websites (http://kobas.cbi.pku.edu.cn/). R language and related packages were used to realize data visualization.

### Statistical analysis

Data were analyzed using SPSS software (version 19.0, Windows, SPSS Inc., Chicago, IL) and Simca software (version 14.1), with a generalized linear model (GLM) to analyze the differences between groups by one-way ANOVA. Statistical significant differences were considered at *P* < 0.05. The graphs were drawn with the GraphPad Prism (version 8.0.2), the R studio (version 4.0.5), and the website (https://biorender.com/).

### Electronic supplementary material

Below is the link to the electronic supplementary material.

Supplementary Material 1

## Data Availability

No datasets were generated or analysed during the current study.
